# The application and prospect of CDK4/6 inhibitors in malignant solid tumors

**DOI:** 10.1186/s13045-020-00880-8

**Published:** 2020-05-01

**Authors:** Qi Du, Xiang Guo, Miao Wang, Yongfu Li, Xiaoyi Sun, Qin Li

**Affiliations:** 1 Department of Oncology, Beijing Friendship Hospital, Capital Medical University, 95 Yongan Road, Beijing, 100050 China; 2 Medical Affairs Department, Pfizer Oncology, Shanghai, 200041 China; 3 Liver research center, Beijing Friendship Hospital, Capital Medical University, Beijing, 100050 China

**Keywords:** CDK4/6 inhibitors, Cell cycle, Tumor signaling pathway, Malignancy, TCGA database

## Abstract

Cyclin-dependent kinase 4/6 (CDK4/6) inhibitors, which block the transition from the G1 to S phase of the cell cycle by interfering with Rb phosphorylation and E2F release, have shown potent antitumor activity and manageable toxicity in HR+/HER2− breast cancer patients. Some clinical trials involving CDK4/6 inhibitors in other tumors have achieved preliminary impressive efficacy. Whether CDK4/6 inhibitors possess great potential as broad-spectrum antitumor drugs and how to maximize their clinical benefits remain uncertain. TCGA database analysis showed that CDK4/6 genes and related genes are widely expressed among various tumors, and high or moderate expression of CDK4/6 genes commonly indicates poor survival. CDK4/6 gene expression is significantly higher in COAD, ESCA, STAD, LIHC, and HNSC, suggesting that CDK4/6 inhibitors could be more efficacious in those tumors. Moreover, network analysis with the STRING database demonstrated that CDK4/6-related proteins were co-expressed or co-occurred with the classical tumor signaling pathways, such as the cell cycle pathway, RAS pathway, PI3K pathway, Myc pathway, and p53 pathway. The extensive antitumor effects of CDK4/6 inhibitors may be achieved by synergizing or antagonizing with other signaling molecule inhibitors, and combination therapy might be the most effective treatment strategy. This article analyzed the feasibility of expanding the application of CDK4/6 inhibitors at the genetic level and further summarized the associated clinical/preclinical studies to collect supportive evidence. This is the first study that presents a theoretical foundation for CDK4/6 inhibitor precision therapy via combined analysis of comprehensive gene information and clinical research results.

Tumorigenesis is a complicated process involving multiple links, multiple factors, and multiple stages, among which the cell cycle plays an essential regulatory role. Dysregulation of the cell cycle is considered to be related to the imbalance of proto-oncogenes, tumor suppressors, and cell cycle-related proteins [1]. Cell cycle inhibitors block cell cycle progression in tumor cells, hence inhibiting tumor cell proliferation and promoting tumor cell apoptosis [2, 3]. To date, cyclin-dependent kinase 4/6 inhibitors (CDK4/6 inhibitors) have occupied the leading position in cell cycle therapy. The successful application of CDK4/6 inhibitors in HR+/HER2− breast cancers brings great clinical benefits to patients and gives more encouragement to physicians and researchers. Since the dysregulation of the cell cycle is one of the crucial characteristics of malignant tumors, we aimed to investigate whether CDK4/6 inhibitors can be applied to various tumors and whether they can be as effective as traditional chemotherapy drugs in most tumors [4].

## The expression of CDK4/6 in tumors

### CDK4/6-related signaling pathways

Cyclin-dependent kinases (CDKs), part of the serine/threonine protein kinase family, are a group of key kinases that regulate the cell cycle; CDKs are activated by cyclins in a time-dependent manner. Twenty kinds of CDKs have been found, and these CDKs bind with their corresponding regulatory subunits (i.e., cyclins) to form active heterodimers [5]. According to their specialized functions, CDKs can be divided into two main categories: CDKs involved in cell cycle regulation, including CDK 1/2/4/6 and CDKs involved in transcriptional regulation, including CDK 7/8/9/11 [6].

**Fig. 1:**
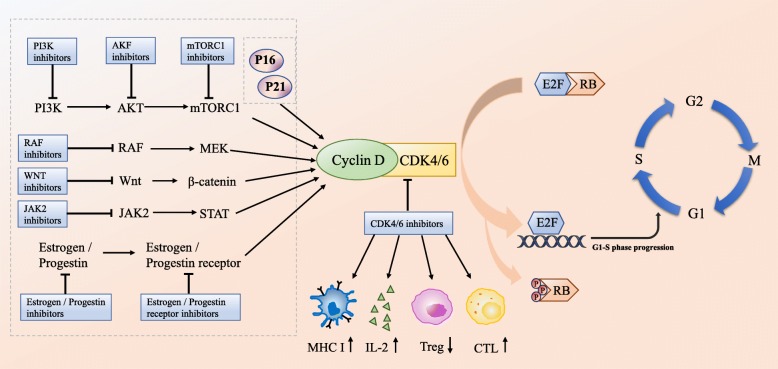
The role of CDK4/6 in the cell cycle

CDK4/6 is the main driving factor in cell cycle regulation and plays a key role in the occurrence and progression of various malignant tumors. Among the four cell cycle phases—G1 phase (prophase of DNA synthesis), S phase (DNA synthesis), G2 phase (prophase of mitosis), and M phase (mitosis)—cyclin D-CDK4/6-retinoblastoma (cyclin D-CDK4/6-Rb) signaling pathway is mainly responsible for regulating the G1-S transition [7]. CDK4 and CDK6 share 71% amino acid homology, and both can bind to cyclin D1/2/3. Under the induction of pro-mitosis signal, cyclin D binds to CDK4/6 and promotes retinoblastoma (Rb) phosphorylation, thus separating transcription factor E2F from the Rb-E2F complex, which causes cells to enter S phase and initiates DNA replication [8, 9] (Fig. 1). Changes in the cyclin D-CDK4/6-Rb pathway have been observed in the tumorigenesis processes of many tumors, such as breast cancer, pancreatic cancer, kidney cancer, liver cancer, and hematologic system tumors [10–17]. Gene amplification, gene mutation, and abnormalities in upstream and downstream regulators of cyclin D, CDK4, and CDK6 can all lead to abnormal activation of the cyclin D-CDK4/6-Rb pathway [8, 18]. The core regulatory effect of CDK4/6 in the cell cycle illustrates its vital role as a target in the treatment of malignant tumors.

### The expression of CDK4/6 in different tumors

**Fig. 2:**
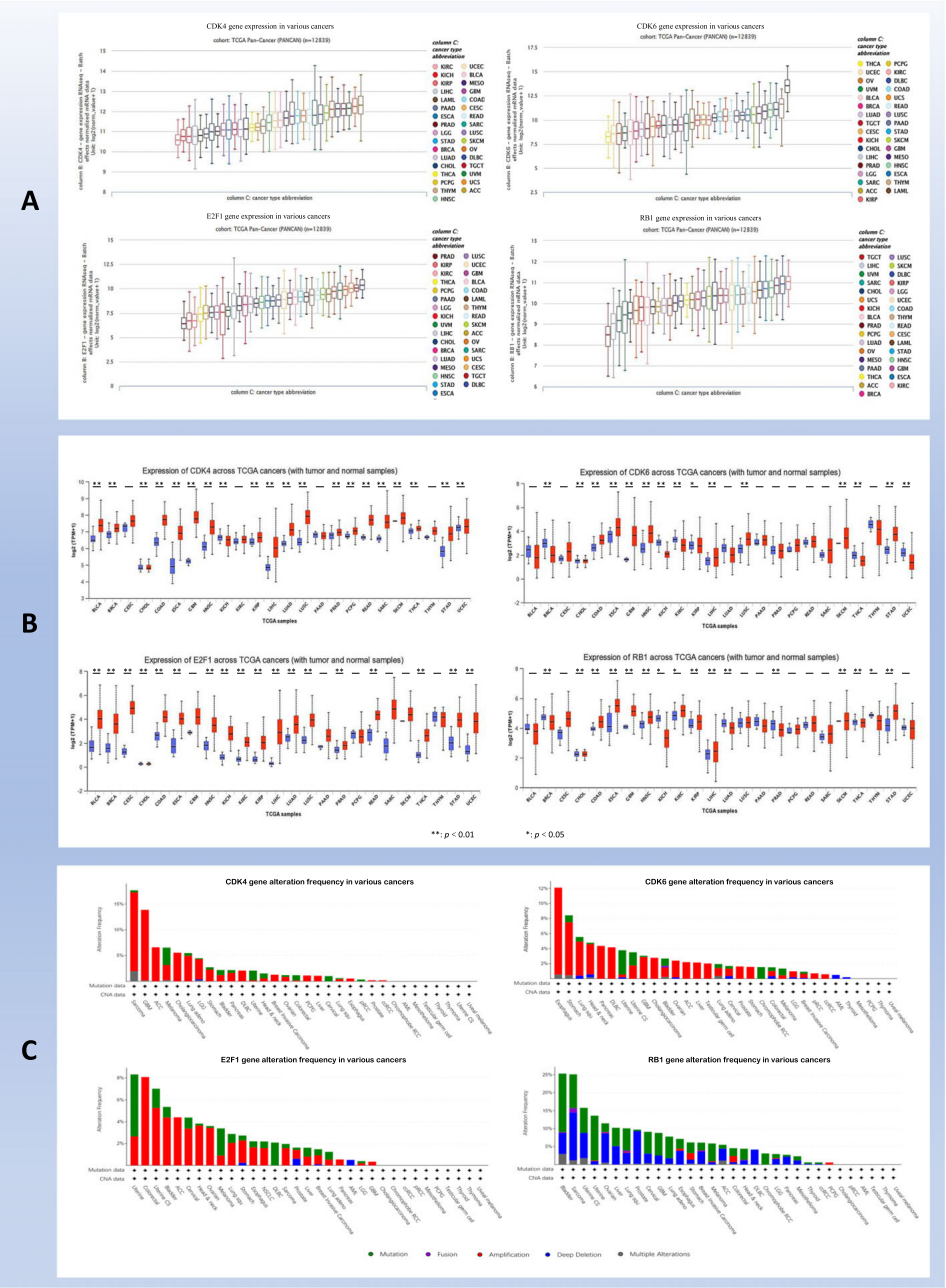
CDK4/6-related gene information among various cancers. **a** Expression of CDK4/6 and related genes. **b** Comparison of the expression of CDK4/6-related genes between tumor and normal tissues. **c** Alteration frequency of CDK4/6-related genes

The *cdk4*, *cdk6*, *rb1*, and *e2f1* genes are expressed in a wide range of cancers according to analysis of the TCGA PANCAN database, which is composed of 12,839 samples. *cdk4* has the highest expression in ACC and the lowest expression in KIRC, *cdk6* has the highest expression in LAML and the lowest expression in THCA, and *e2f1* has the highest expression in DLBC and the lowest expression in PRAD. The expression of *rb1* was the highest in KIRC and the lowest in TGCT. Intriguingly, the expression of *cdk4*, *cdk6*, *e2f1*, and *rb1* in breast cancer was moderate or low compared to that in all tumors. The expression of the four genes in other tumors is shown in Fig. 2a. UALCAN cancer database analysis indicated that the expression of *cdk4* and *e2f1* in breast cancer was significantly higher than that in normal tissues (*p* < 0.01), while the expression of *cdk6* and *rb1* was not higher than that in normal tissues. We ultimately observed that the expression levels of *cdk4*, *cdk6*, *e2f1*, and *rb1* in digestive system tumors such as COAD, ESCA, STAD, LIHC, and HNSC were significantly higher than those in normal tissues (*p* < 0.01), suggesting that CDK4/CDK6-E2F1/Rb1 signaling may be involved in the occurrence and progression of these tumors and that CDK4/6 inhibitors may have better efficacy in these tumors; the expression of *cdk4* and *cdk6* in CESC, PAAD, and THYM was not significantly different compared with that in normal tissues (*p* > 0.05), suggesting that CDK4/6 inhibitors may have poor or no therapeutic effect in these tumors. However, the expression trends of *cdk4*, *cdk6*, *e2f1*, and *rb1* in many other cancers were inconsistent, indicating that this signaling pathway may regulate the cell cycle by crossing with other signaling pathways, and CDK4/6 inhibitor combination therapies may lead to considerable antitumor effects in these cancers (Fig. 2b). Moreover, the *cdk4*, *cdk6*, and *e2f1* genes show extensive gene amplification and gene mutation in various tumors; deep deletion and gene mutation of the *rb1* gene were observed in various tumors, while gene fusion and multiple alterations in these four genes are rare (Fig. 2c). These gene changes may explain the difference in the clinical efficacy of and drug resistance to CDK4/6 inhibitors in different tumors.

### Role of the CDK4/6 gene in tumor progression and prognosis

**Fig. 3:**
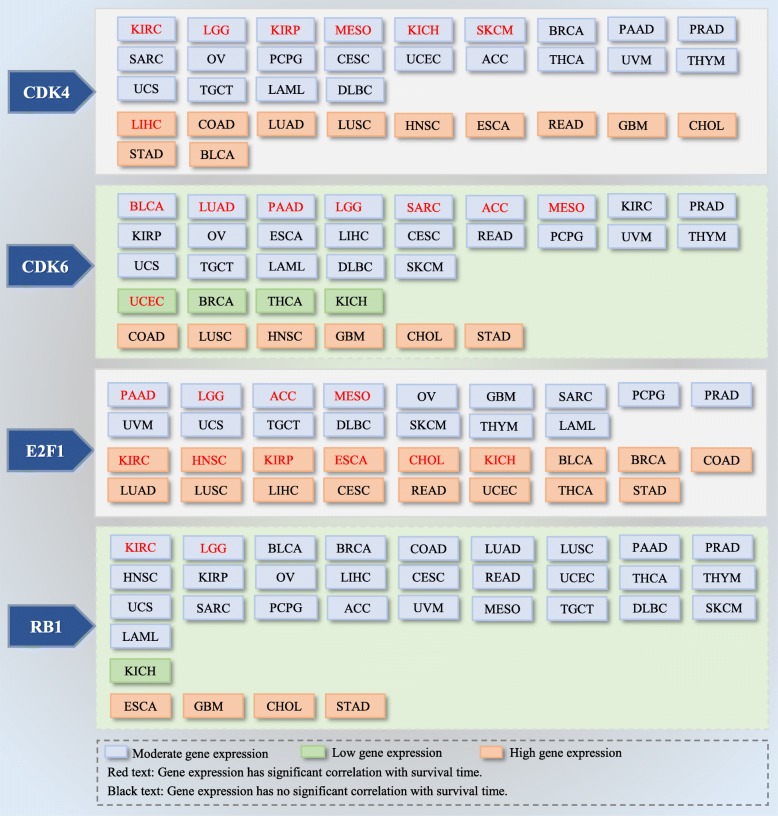
Expression levels of CDK4/6-related genes and its corresponding survival

Based on the analysis from the UALCAN cancer database, moderate expression of the *cdk4* gene in KIRC, LGG, KIRP, MESO, KICH, and SKCM was significantly negatively related to overall survival (*p* < 0.05); high expression of the *cdk4* gene in LIHC was closely related to worse overall survival than low expression and may be a sensitive marker for predicting the prognosis of LIHC. The *cdk6* gene was expressed at low levels in UCEC and moderately expressed in BLCA, LUAD, PAAD, LGG, SARC, ACC, and MESO, and *cdk6* expression was significantly negatively correlated with the overall survival of patients with these tumors. *e2f1* was moderately expressed in PAAD, LGG, ACC, and MESO, and highly expressed in KIRC, HNSC, KIRP, ESCA, CHOL, and KICH, which was closely related to the overall survival of those patients. Moreover, in KIRC and LGG, the *rb1* gene was moderately expressed, and its upregulation predicted a significant decrease in overall survival (Fig. 3). Thus, cdk*4*, *cdk6*, *e2f1*, and *rb1* not only participate in the progression of cancer cells but might also be useful biomarkers for predicting prognosis in some tumors.

## Clinical application of CDK 4/6 inhibitors in breast cancer and other tumors

### Application of CDK 4/6 inhibitors in breast cancer

Based on the essential regulatory role of CDK4/6 in the cell cycle, CDK4/6 inhibitors have emerged as antitumor drugs. CDK4/6 inhibitors hinder the transition from G1 phase to S phase by inhibiting Rb phosphorylation and E2F release and induce tumor cycle arrest at G1 phase, which can inhibit tumor cell growth and cause tumor regression [18]. Since CDK inhibitors were developed 20 years ago, CDK4/6 inhibitors have achieved great success in breast cancer [19, 20], and cell cycle therapy has gradually matured. Approximately, 75–80% of patients with breast cancer are hormone receptor (HR) positive, and the proliferation of breast cancer cells depends on the activation of estrogen [21]. Endocrine therapy is the main treatment for HR positive breast cancer [22]; however, drug resistance is inevitable in the course of treatment [20, 23, 24]. Endocrine therapy combined with chemotherapy is not as effective as expected due to the limited survival benefits and higher toxicity in HR-positive breast cancer patients. Therefore, emphasis should be placed on improving endocrine therapy efficacy [25]. The cyclin D-CDK4/6 complex is usually highly expressed or abnormally activated in breast cancer [19]. The mutation rate of cell cycle-related genes in breast cancer is as high as 38% [26]. Increased expression of cyclin D causes continuous phosphorylation of Rb and leads to continuous proliferation of breast cancer cells; blocking CDK4/6 exerts a lethal effect on breast cancer cells. Moreover, Finn’s study confirmed that palbociclib combined with tamoxifen sensitizes endocrine-resistant estrogen receptor-positive breast cancer cell lines in vitro [27]. Thus, investigators turned their attention to CDK4/6 inhibitors.

Alvociclib, the first-generation CDK inhibitor, lacks specificity and blocks CDK1/2/4/6/7/9, causing serious adverse effects and limiting its clinical application [20]. As medical science has advanced, three selective CDK4/6 inhibitors (palbociclib, ribociclib, and abemaciclib) have achieved fairly good curative benefits in breast cancer [28]. Palbociclib is the first FDA-approved CDK4/6 inhibitor [29]. The PALOMA-1 study showed that palbociclib combined with letrozole increased the PFS of HR+/HER2− advanced breast cancer patients from 10.2 to 20.2 months compared to letrozole alone, and its side effects were controllable [25]. Based on this study, the FDA accelerated the approval of palbociclib in combination with letrozole for first-line treatment of postmenopausal HR+/HER2− metastatic breast cancer patients in February 2015 [25]. The PALOMA-3 study demonstrated that the mOS of the palbociclib combined fulvestrant group was higher than that of the fulvestrant group (34.9 m vs 28.0 m). Accordingly, the FDA approved palbociclib in combination with fulvestrant for HR+/HER2− postmenopausal women with advanced breast cancer who had failed previous endocrine therapy in February 2016 [30]. The phase III study MONALEESA-2 showed that ribociclib plus letrozole significantly improved PFS in patients with HR+/HER2− advanced breast cancer compared with placebo plus letrozole (NR vs 14.7 m; HR 0.56, 95% CI 0.43–0.72, *p* < 0.001) [31]. Based on this trial, ribociclib was approved for first-line treatment of postmenopausal women with HR+/HER2− advanced breast cancer in March 2017 [32]. Palbociclib and ribociclib are breakthroughs in the treatment of advanced HR+/HER2− postmenopausal breast cancer, overturning the old pattern of single endocrine therapy for advanced postmenopausal breast cancer in the past decades, laying the foundation of CDK4/6 inhibitors combined with aromatase inhibitors in first-line treatment for postmenopausal women with HR+/HER2− advanced breast cancer. Abemaciclib is the third FDA-approved CDK4/6 inhibitor. In MONARCH-3 trial, the HR+/HER2− postmenopausal advanced breast cancer patients who did not receive systemic therapy as the aimed population, the 18-month interim results indicated that the PFS of abemaciclib group had not been reached, and the median PFS was 14.7 months in the placebo group; the objective response rates were 59% and 44%, respectively (*p* = 0.004) [33]. According to this study, the FDA approved abemaciclib combined with aromatase inhibitors for the first-line treatment of postmenopausal HR+/HER2− advanced or metastatic breast cancer in February 2018 [34]. Moreover, in the MONARCH-1 trial, the objective response rate of abemaciclib monotherapy reached 19.7% [35]. Compared with palbociclib and ribociclib, abemaciclib can be administered alone, without serious neutropenia toxicity, which provides a new choice for breast cancer patients and highlights its advantages among the highly competitive CDK-targeted drugs. More clinical trial information on the application of CDK4/6 inhibitors in various breast cancers [36–40] is demonstrated in Table 1.

**Table 1:** Clinical trials of CDK4/6 inhibitors in breast cancer

CDK inhibitors	Study ID	Phase	Lines	Patients	Regimens	Efficacy
Palbociclib	PALOMA-1 [25]	II	First-line	HR+/HER2− ABC	Palbociclib + letrozole (*n* = 84)/letrozole (*n* = 81)	mPFS 20.2 m vs 10.2 m, HR 0.488, 95% CI 0.319–0.748, *p* = 0.0004
	PALOMA-2 [36]	III	First-line	HR+/HER2− ABC	Palbociclib + letrozole (*n* = 444)/letrozole (*n* = 222)	mPFS 24.8 m vs 14.5 m, HR 0.58, 95% CI 0.46–0.72, *p* < 0.001
	PALOMA-3 [30]	III	First/second-line	HR+/HER2− ABC with previous ET	Palbociclib + fluvastatin (*n* = 347)/fluvastatin (*n* = 174)	mOS 34.9 m vs 28.0 m, HR 0.81, 95% CI 0.64–1.03, *p* = 0.09
	NCT01684215 [37]	II	First-line	Postmenopausal Japanese patients with HR+/HER2− ABC	Palbociclib + letrozole (*n* = 42)	PFS at 1 year 75.0%, mPFS NR, ORR 40.5%, DCR 85.75%
	NCT02592746 (active, not recruiting)	II	First/second/third-line	Premenopausal Women With HR+ MBC	Palbociclib + exemestane + leuprolide acetate/capecitabine (*N* = 182)	mPFS 9.2 m vs 3.8 m, *p* < 0.001
Ribociclib	MONALEESA-2 [38]	III	First-line	Postmenopausal women with HR+/HER2− ABC	Ribociclib + letrozole (*n* = 334)/letrozole (*n* = 334)	mPFS NR vs 14.7 m, HR 0.56, 95% CI 0.43–0.72, *p* < 0.001
	MONALEESA-3 [39]	III	First/second-line	HR+/HER2− ABC	Ribociclib + fulvestrant (*n* = 484)/fulvestrant (*n* = 242)	mPFS 20.5 m vs 12.8 m, HR 0.59, 95% CI 0.48–0.73, *p* < 0.001
	MONALEESA-7 [40]	III	First/second-line	Premenopausal women with HR+/HER2− ABC	Ribociclib + ET (*n* = 335)/ET (*n* = 337)	mPFS 23.8 m vs 13.0 m, HR 0.55, 95% CI 0.44–0.69, *p* < 0.001
	TRINITI-1 NCT02732119 (active, not recruiting)	I/II	Non-first line	Patients with HR+/HER2− ABC	Ribociclib + everolimus + exemestane (*n* = 107)	mPFS 5.7 m, ORR 8.4%
Abemaciclib	MONARCH-1 [35]	II	Non-first line	Refractory HR+/HER2− ABC	Abemaciclib (*n* = 132)	ORR 19.7%, clinical benefit rate (CR + PR + SD ≥ 6.0 m) 42.4%, mPFS 6.0 m, mOS 17.7 m
	MONARCH-2 [31]	III	Non-first line	HR+/HER2− ABC	Abemaciclib + fulvestrant (*n* = 446)/fulvestrant (*n* = 223)	mPFS 16.4 m vs 9.3 m, HR 0.55, 95% CI 0.45–0.68, *p* < 0.001
	MONARCH 3 [33]	III	First-line	Postmenopausal women with HR+/HER2− ABC	Abemaciclib + nonsteroidal AI (*n* = 223)/nonsteroidal AI (*n* = 223)	mPFS NR vs 14.7 m, HR 0.54, 95% CI 0.41–0.72, *p* < 0.001, ORR 59% vs 44%, *p* = 0.004

*ABC* advanced breast cancer, *MBC* metastatic breast cancer, *ET* endocrine therapy, *AI* aromatase inhibitor, *NR* not reached, *PFS* progression-free survival, *OS* overall survival, *ORR* objective response rate, *DCR* disease control rate, *HR* hazard ratio

The major adverse effects of CDK4/6 inhibitors are leukopenia and neutropenia, mainly caused by palbociclib and ribociclib [41]. CDK4/6 inhibitors can also cause gastrointestinal side effects such as diarrhea, nausea, and vomiting. It is worth noting that once neutropenia occurs simultaneously with diarrhea, the risk of infection is greatly increased [42]. Some patients have prolonged QTc intervals, elevated transaminases, thromboembolism, and others [43]. However, these side effects are reversible and can be controlled by dose interruption, dose reduction, and symptomatic supportive treatment [44].

### Application and expansion of CDK4/6 inhibitors in other solid tumors

The *cdk4/6* genes are generally expressed among various cancers, and their expression is higher or lower than that of normal tissues in most cancer types. High or moderate expression of CDK4/6-related genes often indicates poor survival. The CDK4/6 genes participate in tumorigenesis by synergistically regulating multiple genes in many signaling pathways. CDK4/6 inhibitors may be more effective when combined with other signaling pathway inhibitors, which is consistent with the complexity and intersectionality of tumor signaling pathway interactions. Although the *cdk4/6* gene expression level in breast cancer is relatively low, a marked antitumor effect of CDK4/6 inhibitors has been observed in breast cancer, so we speculate that it may also work in other malignancies, especially in those cancers with high cdk4/6-related gene expression. CDK4/6 inhibitors may have the potential to be widely applied in multiple cancers, similar to traditional chemotherapy drugs such as cisplatin or paclitaxel.

Although main battlefield in HR+/HER2− breast cancer, CDK4/6 inhibitors have also been actively explored in other malignancies. Yang’s study showed that CDK4/6 inhibitors increased the sensitivity of acute myeloid leukemia cells to cytotoxic drugs [17]. Patnaik and Taylor’s study showed that CDK4/6 inhibitors achieved disease control rates of 49% and 44%, respectively, in non-small-cell lung cancer patients (*n* = 68) and melanoma patients (*n* = 18) [44, 45]. Adkins’ study indicated an objective response rate of 39% for CDK4/6 inhibitors in patients with head and neck squamous cell carcinoma (*n* = 62) [46]. It is not difficult to conclude that the exploration of CDK4/6 inhibitors in other cancers except breast cancer, such as liposarcoma, lymphoma melanoma, and many other advanced cancers [47–50], is still at an early stage and is mostly limited to basic experiments and stage I or II clinical trials (See Table 2).

**Table 2:** Clinical trials of CDK4/6 inhibitors in other tumors

CDK inhibitors	Study ID	Phase	Lines	Patients	Regimens	Efficacy
Palbociclib	NCT01209598 [47]	II	Non-first line	Advanced WD/DDLS	Palbociclib (*n* = 60)	PFS at 12 weeks 57.2%; mPFS 17.9 weeks
	NCT02101034 [46]	II	Non-first line	HNSCCs	Palbociclib + cetuximab (*n* = 62)	ORR 39% (in platinum-resistant patients), ORR 19% (in cetuximab-resistant patients)
	NCT01037790 (recruiting)	II	UK	RB/germ cell tumors	Palbociclib (*n* = 205)	PFS at 6 months 28%; mPFS 11 weeks
	NCT01536743 (active, not recruiting)	II	Non-first line	Ovarian epithelial carcinoma	Palbociclib (*n* = 26)	PFS at 6 months 15%
	NCT00420056 [48]	Ib	UK	MCL	Palbociclib (*n* = 17)	6% CR, 12% PR, 41% SD, mPFS 4.0 m
Ribociclib	CLEE011X2105 [45]	Ib/II	Non-first line	BRAF V600-mutant melanoma	Ribociclib (*n* = 18)	2 PR, 6 SD
	CMEK162X2114 [49]	Ib/II	UK	NRAS-mutant melanoma	Ribociclib + binimetinib (*n* = 22)	7 PR, 11 SD, 33% had 20–30% tumor shrinkage
Abemaciclib	NCT01394016 [44]	I	UK	Breast cancer; NSCLC; Melanoma; Glioblastoma; CRC	Abemaciclib (*n* = 225)	Breast cancer (*n* = 47) 23% PR, 47% SD, 23% ORR, 49% CBR, 70%DCR, mPFS 5.8 m; NSCLC (*n* = 68), 3% PR, 46% SD, 3% ORR, 49% DCR, mPFS 2.0 m; melanoma (*n* = 26) 4% PR, 23% SD, 4% ORR, 27% DCR; glioblastoma (*n* = 17) 18% SD, 18% DCR; CRC (*n* = 15) 13% SD, 13% DCR
	NCT02014129 [50]	I	Non-first line	Various advanced cancer	Abemaciclib (*n* = 12)	tumor size changed from 35% decrease to 25% increase, > 30% tumor shrinkage in 2 patients

*WD/DDLS* well-differentiated or dedifferentiated liposarcoma, *HNSCCs* head and neck squamous-cell carcinomas, *Rb* retinoblastoma, *MCL* mantle cell lymphoma, *NSCLC* non-small-cell lung cancer, *CRC* colorectal cancer, *UK* unknown, *PFS* progression-free survival, *OS* overall survival, *ORR* objective response rate, *DCR* disease control rate, *CR* complete response, *PR* partial response, *SD* stable disease, *HR* hazard ratio

## Correlations between CDK4/6-related proteins and classical tumor signaling pathway molecules

**Fig. 4:**
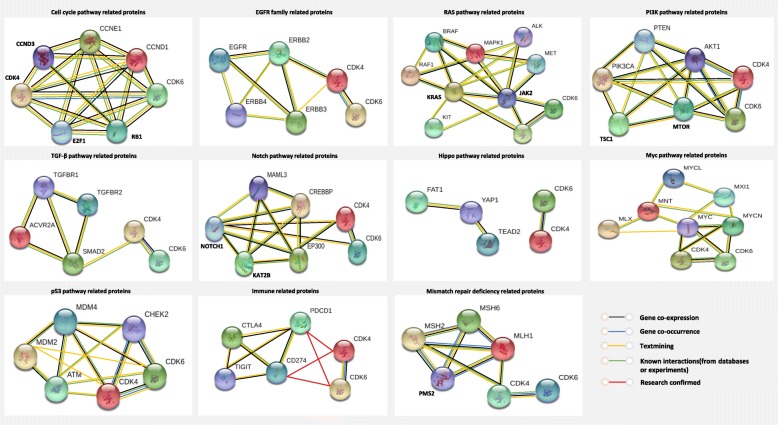
Functional protein association networks of CDK4/6 and classical tumor signaling pathways

The occurrence and progression of tumors involves the interaction of multiple signaling pathways. We used the STRING database and the STRING tool to map the relationship between CDK4/6-Rb1/E2F1 pathway proteins and classical signaling pathway-associated proteins. Network analysis showed that CDK4/6-related proteins were directly or indirectly associated with key molecules of classical signaling pathways, such as the cell cycle pathway, RAS pathway, PI3K pathway, Myc pathway, and p53 pathway. In addition, CDK4/6 protein is co-expressed with EGFR/ERBB2, BRAF/KRAS, PIK3CA/MTOR, MYC/MYCN, NOTCH, MDM4, MSH2, and other proteins (Fig. 4, black line), suggesting that CDK4/6 may have synergistic effects with these co-expressed signaling molecules. Moreover, CDK4/6-related signaling molecules are downstream of most signaling pathways, such as the RAS pathway/PI3K pathway/TGF-β pathway/p53 pathway/Notch pathway/Myc pathway [51], suggesting that CDK4/6 blockade may inhibit these related signaling pathways to some extent. There were also protein correlations derived from experimental evidence (Fig. 4, green line) and some derived from in-depth analysis of scientific papers (Fig. 4, yellow line). CDK4/6 inhibitors may play a role in inhibiting tumor growth by synergizing or antagonizing certain signaling molecule inhibitors and immune checkpoint inhibitors, which indirectly reflects their extensive antitumor effect.

### CDK4/6 inhibitors combined with endocrine therapy

At present, endocrine therapy combined with CDK4/6 inhibitors has achieved significant therapeutic effects and controllable toxicity in many clinical trials, as mentioned above, and combined therapy has become the most promising therapeutic strategy for HR+/HER2− breast cancer patients.

### CDK4/6 inhibitors combined with immunotherapy

CDK4/6 inhibitors not only arrest the tumor cell cycle, but also trigger antitumor immunity. First, CDK4/6 inhibitors downregulate E2F transcription factor-related gene expression and upregulate major histocompatibility complex class I molecule expression in breast cancer cell lines [52]; CDK4/6 inhibitors activate endogenous retroviral components in tumor cells, stimulating the production of type III interferons to promote tumor antigen presentation [52, 53]. Second, CDK4/6 inhibitors inhibit the proliferation of regulatory T (Treg) cells and DNA methyltransferase 1 expression in Treg cells, resulting in promoter hypomethylation and suppression of E2F release [52]; other studies demonstrated that the expression of CDK6 in Treg cells was higher than that of other T cell subtypes. CDK4/6 inhibitors can downregulate Treg cell proliferation by inhibiting CDK6 [54]. Furthermore, CDK4/6 inhibitors promote tumor cell clearance by enhancing cytotoxic T cells (CTLs). CDK6 is an upstream regulatory element of nuclear factor of activated T cells (NFAT), and CDK4/6 inhibitors suppress NFAT phosphorylation, the activation of CTLs, and its ability to kill tumor cells [55]. Finally, the Cyclin D1-CDK4 complex directly phosphorylates speckle-type POZ protein (SPOP), and CDK4/6 inhibitors can enhance the immune escape of tumors by decreasing the ubiquitination of SPOP and reducing the degradation of PD-L1 [56, 57]. These synergistic mechanisms provide a theoretical basis for the combination therapy of CDK4/6 inhibitors plus immune checkpoint inhibitors [52, 58]. In Zhang’s study, CDK4/6 inhibitor combined with PD-1 antibody significantly reduced the proliferation of tumor cells and improved the survival rate of carcinogenic mic e[57], illustrating the synergistic antitumor effect of the CDK4/6 inhibitor and immune checkpoint-related inhibitor. The synergism, antagonism, and interactions of those related genes deserve further exploration (Fig. 4, red line).

### CDK4/6 inhibitors combined with targeted therapy

The PI3K-AKT-mTOR pathway proteins and the CDK-Rb-E2F pathway proteins are widely co-expressed in different tumor types, and the two pathways are the relationship between the upstream and downstream, signifying that the combined targeting inhibition of CDK4/6 and PI3K-AKT-mTOR should further improve the efficacy [59]. Cortes’ research indicated that the combination of PI3K inhibitors or mTOR inhibitors might increase the sensitivity of CDK4/6 inhibitors [60]. Teo’s study showed that CDK4/6 inhibitors combined with PI3K inhibitors increased the apoptosis of triple-negative breast cancer cell lines and induced persistent tumor regression in vivo [61]. Moreover, CDK4/6 inhibitors can restore sensitivity to EGFR/HER2 inhibitors by reducing the activity of mTOR [62]. EGFR/HER2 inhibitors combined with CDK4/6 inhibitors may increase the sensitivity to EGFR inhibitor-resistant lung cancer cells [63]. Goel’s study demonstrated that CDK4/6 inhibitors augmented the efficacy of EGFR inhibitors in esophageal squamous cell carcinoma and reversed drug resistance [64]. A phase II clinical trial (NCT02101034) involving patients with HPV-unrelated head and neck squamous-cell carcinomas showed that palbociclib plus cetuximab resulted in an objective response rate of 39% in patients with disease progression on platinum but cetuximab-naive and 19% in patients with disease progression on cetuximab [46]. Teh’s study demonstrated that CDK4/6 inhibitors in combination with BRAF inhibitors or MEK inhibitors are an effective treatment strategy for melanoma, but continued administration increases toxicity. The mechanism of acquired resistance of CDK4/6 inhibitors combined with BRAF inhibitors or MEK inhibitors may be due to mTOR pathway activation, so adding mTOR inhibitors may overcome the resistance of foresaid combined therapy [65]. Chen’s study showed that RAF inhibitors combined with CDK4/6 inhibitors improve the therapeutic effects of RAS or BRAF mutant tumors [66]. Small’s study confirmed that abemaciclib combined with sunitinib significantly decreased tumor size in a preclinical mouse model of renal cell carcinoma without serious adverse effects [67]. Pek’s study demonstrated that in KRAS-dependent and BRAF-mutant metastatic colorectal cancer cell lines, palbociclib combined with MEK inhibitors is an effective treatment strategy [68]. Ribociclib combined with the ALK inhibitor ceritinib showed excellent therapeutic effects in ALK mutant neuroblastoma [69]. However, large-scale clinical trials are needed to confirm the efficacy of the abovementioned combination therapies.

### CDK4/6 inhibitor combined with other treatments

Cyclin D3-CDK6 can phosphorylate two key enzymes (6-phosphofructokinase and pyruvate kinase M2) in the glucose metabolism pathway and restrain its metabolic activity, which consumes the antioxidants NADPH and glutathione, therefore increasing the level of reactive oxygen species and leading to apoptosis of tumor cells. Palbociclib combined with low-dose pentose phosphate pathway inhibitors may exert synergistic antitumor effects [70]. Vijayaraghavan’s research showed that CDK4/6 inhibitors combined with autophagy inhibitors can maintain the integrity of the G1/S checkpoint and may be a new therapeutic pattern for multiple solid tumors [71]. Francis’s study confirmed that CDK4/6 inhibitors resensitize Rb-positive sarcoma cells to the Weel kinase inhibitor AZD1775 [72].

## The resistance and efficacy prediction of CDK4/6 inhibitors

CDK4/6 inhibitor resistance inevitably occurs in the course of treatment, and overcoming resistance is a challenge for clinicians. Some studies have pointed out that the acquired resistance of CDK4/6 inhibitors mainly results from *CDK6* amplification, which usually occurs after prolonged administration of the potent selective CDK4/6 inhibitor [73]. Some researchers believe that *CCNE1* amplification causes acquired resistance to CDK4/6 inhibitors, and that sensitivity can be restored by targeting CDK2 [74]. In addition, activating *cyclin D* gene mutations may enhance sensitivity to CDK4/6 inhibitors, while *cyclin D* deficiency is associated with CDK4/6 inhibitor resistance [75]. In addition, the role of CDK4/6 inhibitors in arresting the cell cycle depends on *Rb* status [24]. When *Rb* function is absent, the G1-S phase transition is no longer dependent on CDK4/6, and CDK4/6 inhibitors may have off-target effects in *Rb* inactivation models [19]. By influencing the Hippo pathway, *FAT atypical cadherin 1* (FAT1) loss leads to CDK6 elevation, which promotes resistance to CDK4/6 inhibitors [76]. A recent study revealed that amplification of *fibroblast growth factor receptor 1* (FGFR1) might cause resistance to CDK4/6 inhibitors [77]. We can preliminarily conclude that the increase in cyclin-CDK complex expression and the regulation of the cyclin D-CDK4/6-Rb pathway may be important resistance mechanisms of CDK4/6 inhibitors, as shown in Table 3. Overcoming resistance is a major concern in research, and exploring efficacy predictors and selecting the population that should obtain the most benefit is another research hot spot. Studies have shown that deletion of *p16* decreases the endogenous inhibition of CDK4/6 and that low levels of *p16* may suggest that cells are sensitive to CDK4/6 inhibitors [78]. High expression of *cyclin D*/*Rb* and low expression of *p16* are thought to be biomarkers for predicting CDK4/6 inhibitor sensitivity [27]. However, Wang’s study showed that approximately 85% of breast cancer cells have normal *Rb* status, but due to the rare *Rb* deletion in ER+ breast cancer, it is less sensitive as a predictive marker [79]. CDK activation requires interferon β expression, suggesting that interferon β may be a predictor of CDK4/6 inhibitor efficacy [80]. Other efficacy predictors include CDK4 phosphorylation and tumor cloning kinetics [75, 81, 82]. However, so far, there is still a long way to go in combatting CDK4/6 inhibitor resistance and identifying sensitive predictive markers.

**Table 3.** Factors contributing to resistance to CDK4/6 inhibitors.

Resistance type	Resistance mechanism
Cyclin-CDK complex increase	*CDK6* amplification
	*CCNE1* amplification
Cyclin D-CDK4/6-Rb pathway regulation	*Cyclin D* deficiency *Rb* inactivation or deletion
	*FAT1* loss
Others	*FGFR1* amplification

## Prospects and conclusions

CDK4/6-related genes are widely expressed and are closely associated with the prognosis of different cancers. CDK4/6 inhibitors achieve striking antitumor effects by regulating the cell cycle. Clinical trials have confirmed that CDK4/6 inhibitors alone or combined with endocrine therapy have brought great clinical benefit to HR+/HER2− breast cancer patients. Extensive cross-linking of CDK4/6 genes and key genes in classical tumor signaling pathways may exert synergistic or antagonistic effects, given the theoretical basis for CDK4/6 inhibitor combination therapy. CDK4/6 inhibitors have great potential to become broad-spectrum antitumor drugs. CDK4/6 inhibitor combination therapy may become a new strategy for the precise treatment of malignancies. Future research should focus on exploring biomarkers that predict CDK4/6 inhibitor efficacy and screening sensitive populations. More large-scale prospective clinical trials are needed to validate the curative effect of CDK4/6 inhibitors in more cancer types and more combination models.

## Data Availability

All data generated or analyzed during this study are included.

## Abbreviations

TCGA: The Cancer Genome Atlas
ACC: Adrenocortical carcinoma
BLCA: Bladder urothelial carcinoma
BRCA: Breast invasive carcinoma
CESC: Cervical squamous cell carcinoma and endocervical adenocarcinoma
CHOL: Cholangiocarcinoma
COAD: Colon adenocarcinoma
DLBC: Lymphoid neoplasm diffuse large B-cell lymphoma
ESCA: Esophageal carcinoma
GBM: Glioblastoma multiforme
HNSC: Head and neck squamous cell carcinoma
KICH: Kidney chromophobe
KIRC: Kidney renal clear cell carcinoma
KIRP: Kidney renal papillary cell carcinoma
LAML: Acute myeloid leukemia
LGG: Brain lower grade glioma
LIHC: Liver hepatocellular carcinoma
LUAD: Lung adenocarcinoma
LUSC: Lung squamous cell carcinoma
MESO: Mesothelioma
OV: Ovarian serous cystadenocarcinoma
PAAD: Pancreatic adenocarcinoma
PCPG: Pheochromocytoma and paraganglioma
PRAD: Prostate adenocarcinoma
READ: Rectum adenocarcinoma
SARC: Sarcoma
SKCM: Skin cutaneous melanoma
STAD: Stomach adenocarcinoma
TGCT: Testicular germ cell tumors
THCA: Thyroid carcinoma
THYM: Thymoma
UCEC: Uterine corpus endometrial carcinoma
UCS: Uterine carcinosarcoma
UVM: Uveal melanoma
STES: Stomach and esophageal carcinoma
EGFR: Epidermal growth factor receptor
PI3K: Phosphatidylinositol 3 kinase
TGF-β: Transforming growth factor beta
MTOR: Mammalian target of rapamycin
MAPK: Mitogen-activated protein kinase
PD-1: Programmed cell death-1
PD-L1: Programmed cell death-ligand 1
MSH: Mismatch repair
